# Involvement of an Enhanced Immunity Mechanism in the Resistance to *Bacillus thuringiensis* in Lepidopteran Pests

**DOI:** 10.3390/insects14020151

**Published:** 2023-02-01

**Authors:** Zeyu Xiao, Xue Yao, Sufen Bai, Jizhen Wei, Shiheng An

**Affiliations:** State Key Laboratory of Wheat and Maize Crop Science, College of Plant Protection, Henan Agricultural University, Zhengzhou 450002, China

**Keywords:** *Bacillus thuringiensis*, immune response, immune priming, resistance mechanism, resistance management

## Abstract

**Simple Summary:**

Immune responses of Lepidopteran pests to *Bacillus thuringiensis* (*Bt*) or *Bt* toxins, including pattern recognition proteins, antimicrobial peptides (AMPs) and their synthetic signaling pathways, the prophenoloxidase system, reactive oxygen species (ROS) generation, nodulation, encapsulation, phagocytosis, cell-free aggregates, contribute to the evolution of insect resistance to *Bt*. Targeting the insect immune response and resistance to *Bt* or *Bt* toxins may help to improve insecticidal activity and manage insect resistance.

**Abstract:**

*Bacillus thuringiensis* (*Bt*) is the safest, economically successful entomopathogen to date. It is extensively produced in transgenic crops or used in spray formulations to control Lepidopteran pests. The most serious threat to the sustainable usage of *Bt* is insect resistance. The resistance mechanisms to *Bt* toxins depend not only on alterations in insect receptors, but also on the enhancement of insect immune responses. In this work, we review the current knowledge of the immune response and resistance of insects to *Bt* formulations and *Bt* proteins, mainly in Lepidopteran pests. We discuss the pattern recognition proteins for recognizing *Bt*, antimicrobial peptides (AMPs) and their synthetic signaling pathways, the prophenoloxidase system, reactive oxygen species (ROS) generation, nodulation, encapsulation, phagocytosis, and cell-free aggregates, which are involved in immune response reactions or resistance to *Bt*. This review also analyzes immune priming, which contributes to the evolution of insect resistance to *Bt*, and puts forward strategies to improve the insecticidal activity of *Bt* formulations and manage insect resistance, targeting the insect immune responses and resistance.

## 1. Introduction

*Bacillus thuringiensis* (*Bt*) is a Gram-positive bacterium with entomopathogenic properties that can produce spores and insecticidal proteins [including Crystal (Cry), Cytolytic (Cyt) proteins, and vegetative (Vip) insecticidal proteins]. It is widely used to control Lepidopteran pests, Coleoptera, Diptera, and nematodes by spraying *Bt* formulations or planting transgenic crops (expressing *Bt* proteins). *Bt* formulations are considered more specific and safer than chemical pesticides. They are used in the vast majority of formulated sprayable bacterial microbial pesticides [1,2,3,4], which contain viable *Bt* spores and Cry toxins as active ingredients [5]. Moreover, *Bt* crops that express *Bt* proteins have been extensively planted in 29 countries, accounting for a global area of more than 108 million hectares worldwide, which represents more than 53% of the global planted area of transgenic crops [6].

Due to the commercial interest in *Bt* formulations and *Bt* crops, the exploration of their modes of action has received considerable attention. As an entomopathogenic bacterium, the killing mechanisms of *Bt* involve the pore-forming toxins induced by *Bt* proteins [7,8,9,10,11] and septicemia induced by *Bt* or midgut bacteria [12,13,14,15,16]. Furthermore, one of the unavoidable outcomes of the continual and extensive pest control with *Bt* formulations and *Bt* crops has been the emergence of insect tolerance or resistance to *Bt* [17,18,19,20]. However, researchers of host resistance mechanisms to *Bt* are primarily concerned about the genetic resistance to *Bt* toxins, as they induce a high level of resistance based on mutations of target sites or the reduction of receptor abundance on the midgut epithelium [10,21,22,23]. Indeed, the host immune responses could be activated by *Bt* and *Bt* proteins to counteract the toxic effects [20,24,25]. In particular, the inducible low-level resistance or tolerance, which may be caused by gene and protein regulatory mechanisms, could be related to the relative activities and amounts of immune components, thereby resulting in the sequestration or inactivation of the toxin [26,27,28,29,30,31]. Importantly, insects show a phenomenon called “immune priming”, wherein insects can prolong the activation of immune responses and transmit their immune status to the next generation. This immune priming could cause the evolution of resistance acquired upon repeated individual infections or from immune-challenged parent(s) [29].

*Bt* formulations and *Bt* crops are mainly used to control agriculture pests, especially, Lepidopteran pests. These pest insects have a robust immune system based on hemocytes, antimicrobial peptides (AMPs), phenoloxidase (PO), lysozyme, nodulation, encapsulation, phagocytosis, and other mechanisms acting in different immune pathways, which are activated in response to infection [26,27,28,29,30,31,32,33,34], including that caused by the *Bt* bacterium and *Bt* proteins [20,25,31,35]. In addition, changes in immune-related genes or proteins in the above immune pathways participate in insect resistance to *Bt* bacterium or *Bt* proteins [26,27,28,29,30,31,36,37,38,39]. For example, Ma et al. reported that laboratory *Bt*-resistant strains of *Helicoverpa armigera* showed increased melanization and coagulation reaction (due to high levels of a soluble toxin-binding glycoprotein), and this resistance mechanism against toxins based on a systemic immune induction could be transmitted to the next generation by a maternal effect [26]. Currently, the response mechanisms and immune resistance of insects is attracting worldwide attention to improve the insecticidal activity of *Bt* and managing insect resistance to *Bt*. In fact, understanding the host response mechanisms and immune resistance to *Bt* and *Bt* proteins will enable us to identify more ways of pest control and resistance management [40,41,42,43,44,45]. In this review, we will focus on the involvement of enhanced immune defense of insects against *Bt*, including the pattern recognition proteins for recognizing *Bt*, AMPs and their synthetic signaling pathways, the prophenoloxidase system, ROS generation, nodulation, encapsulation, phagocytosis, and cell-free aggregates. Importantly, on the basis of these immune responses and resistance, we provide some suggestions to improve *Bt* insecticidal activity and manage insect resistance.

## 2. Immunity Induced by *Bt*

Researchers confirmed that *Bt* has specific effects on insect immunity when administered either orally or by injection [28,31,46]. The immune barriers include the gut lumen, but mainly consist in the hemocoel that *Bt* spores, toxins, and enteric microbes could enter through midgut lesions that are caused by *Bt* toxins [12,13,14,15,16]. The insects depend on their innate immunity to quickly recognize and destroy or immobilize invasive pathogens. The immune responses comprise humoral and cellular factors [47,48]. The humoral component includes the synthesis of AMPs, PO-mediated melanization, bacteriolytic enzymes, the production of proteases, heat shock proteins, and lectins [49,50,51,52,53,54,55]. Hemocytes are key elements in cellular immune responses and are involved in the killing of pathogens through the production of pattern recognition proteins/receptors (PRPs), proteins responsible for nodulation, encapsulation, phagocytosis, and other molecules (AMPs, melanization modulators and enzymes, stress response proteins, and signal transduction proteins) that are involved in the killing of pathogens [56]. As reported, sublethal doses of *Bt* widely induced the humoral and cellular immune response in insects, even leading to immune resistance to *Bt* [27,28,47,48,57,58].

### 2.1. Recognition of B. thuringiensis

In both humoral or cellular immunity, the first step in the initiation of the immune response is pathogen recognition. This step is achieved by the recognition of and the interaction between pathogen-associated molecular patterns (PAMPs) located on the surface of the pathogen and PRPs [59,60,61]. The most commonly characterized PRPs are peptidoglycan-recognizing proteins (PGRPs), C-type lectins, scavenger receptor, and apolipophorin III (ApoLp-III) [62,63,64]. It is reported that the expression of PGRPs in *Plutella xylostella* and *Ostrinia furnacalis* was induced to activate the immune response to *Bt* [65,66]. The silencing of PxPGRP-S1 by RNA interference (RNAi) significantly reduced the expression of AMP, which caused high mortality under *Bt* treatment [67]. The scavenger receptor C can bind the *Bt* bacterium to enhance AMP expression in hemocytes via the Toll pathway and protect *Bombyx mori* from *Bt* pathogens [36].

ApoLp-III is a pattern recognition protein that participates in humoral immune reactions against invading pathogens [68], activates the PPO cascade [69,70], induces antibacterial activity [37], and works in cellular immune reactions to stimulate encapsulation and phagocytosis [68,71]. Meanwhile, an ApoLp precursor of *Diatraea saccharalis* in a resistant strain of Cry1Ab was significantly upregulated compared with its levels in a susceptible strain [72]. The ApoLp-III protein was also upregulated under treatment with spore–crystal mixtures of *Bt* strains producing Cry3Ba toxins in *Tribolium castaneum* (Tc) to regulate PO enzyme activity [73].

C-type lectins are PRPs involved in innate immunity in invertebrates. They play important roles in pathogen recognition, AMP synthesis, melanization, and encapsulation or in the direct killing of bacteria [74,75,76,77,78,79]. C-type lectins from *H. armigera* can agglutinate *Bt* in the presence of Ca^2+^ and inhibit the growth of *Bt* in vivo by increasing hemocyte phagocytosis [80]. In *T. castaneum*, C-type lectins also bind to *Bt* toxins in the presence of Ca^2+^, playing a key role in the immune response toward pathogen infection by affecting the expression of AMPs and the agglutination of bacteria in the presence of Ca^2+^ [81]. In *P. xylostella*, C-type lectins can bind to the PAMPs of *Bt* 8010 and mediate innate immunity, e.g., by enhancing the adsorption ability of hemocytes and PO activity and melanization [82].

### 2.2. Antimicrobial Peptides and Their Synthetic Signaling Pathways Participate in Fighting Bt

Once a foreign element is detected by PRPs, a series of signaling molecules are activated. One of the first insect defense mechanisms identified was the generation of AMPs via the Toll and IMD pathways [83,84]. In response to *Bt* or *Bt* proteins infection, the expression of AMPs (gloverin, moricin, lebocin, attacin, cecropins, cobatoxin A, etc.) is induced in several insects [24,38,39,66,85,86,87,88,89,90]. Insect resistance to *Bt* is also reportedly associated with insect AMPs [91,92]. In *S. exigua* and *Galleria mellonella*, RNAi-mediated knockdown of gloverin enhanced the susceptibility to *Bt* [39,93]. AMPs may act in the immune responses and resistance to *Bt* by killing the bacteria or blocking their growth, thus clearing *Bt* from the midgut [39,93,94,95,96]. After exposure to *Bt*, the surviving resistant *G. mellonella* with higher levels of AMPs (specifically, of gloverin) successfully purged *Bt* from the midgut, and ~30% of the perished insects contained *Bt* subpopulations that were blocked in two stages, namely, necrotrophy and sporulation [39]. Other mechanisms mediated by AMPs in the immune response and resistance to *Bt* may help to screen and establish certain flora in the gut or kill the bacteria that enter the humoral system after all the host microbiota has participated in septicemia during infection with *Bt* [39,96,97]. The composition and activities of the gut microbiota that are linked to host immunity and *Bt* toxicity have been well reviewed [31].

**Figure 1:**
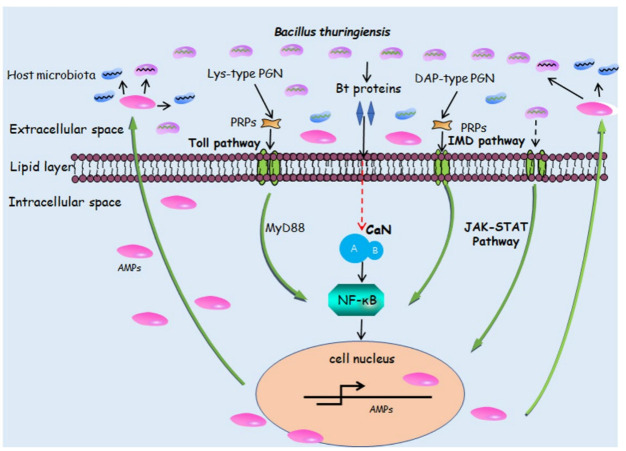
A simplified overview of innate immunity mechanisms activating synthetic signaling pathways of AMPs induced by *Bacillus thuringiensis* and/or its toxins. Toll pathway, IMD pathway, JAK–STAT pathway, and calcineurin (CaN) pathway promote the production of antimicrobial peptides (AMPs) to counter *B. thuringiensis* and/or its toxins. PGN: peptidoglycan; NF-κB: nuclear factor-κB; PRPs: pattern recognition proteins.

AMPs are synthesized via the Toll, Imd, and JAK/STAT pathways (Figure 1) [84]. Generally, insects respond to Gram-positive bacterial and fungal infections through the Toll pathway, while Gram-negative bacteria activate AMP synthesis through the Imd pathway [98]. MyD88 acts downstream of Toll to activate the translocation of Dorsal or Dorsal-related immune factors (which belong to the nuclear factor-κB family) into the nucleus to activate the transcription of AMPs [83]. MyD88 has been reported to participate in regulating the expression of AMPs to resist *Bt* infection in *O. furnacalis* larvae [38]. Knocking down Dorsal expression in *P. xylostella* increaseed the susceptibility of *P. xylostella* larvae to live *Bt* [99]. However, studies showed that Dorsal could bind to Cry1Ab1 toxin in *P. xylostella* and *S. exigua* [100]. How these bindings affect AMP expression and the insecticidal activities of *Bt* toxins remain unclear.

Interestingly, the wall of *Bacillus* spp. also contains DAP-type peptidoglycan (PGN), typical for Gram-negative bacterium, and the Imd pathway is induced by *Bt* (Figure 1) [66,101,102]. In the Imd pathway, signal transduction causes the cleavage of Relish, and the rel domain translocates to the nucleus to activate AMP synthesis [98]. The JAK–STAT pathway can control the production of AMPs in the insect midgut and promote the repair of insect midgut epithelial cells destroyed by *Bt*8010 [66,103].

In many insects, Cry or Vip toxins alone can kill insect larvae (used in transgenic crops that express the activated Cry or Vip toxins), and the Cry or Vip toxins could also trigger the transcriptional activation of AMPs [35]. Additionally, when heat-killed *Bt* suspension cells were injected into the *B. mori* larval hemocoel, AMPs including attacin, gloverin, lebocin, and moricin, were found to be significantly upregulated [24]. However, how do *Bt* proteins induce AMP expression? It was reported that *Drosophila* calcineurin (CaN, Ca^2+^-dependent phosphatase) promotes the induction of innate immunity through Toll and Imd (Figure 1) [104]. CaN serves as an immune activator by interacting with Relish to regulate AMP (gloverin, cecropin D, and attacin) expression in *H. armigera* (Figure 1) [105]. Importantly, CaN activity could be induced by Cry1Ac and Cry2Ab [40,41]. Consequently, AMP expression was promoted. 

### 2.3. Melanization Reaction for Fighting Bt

**Figure 2:**
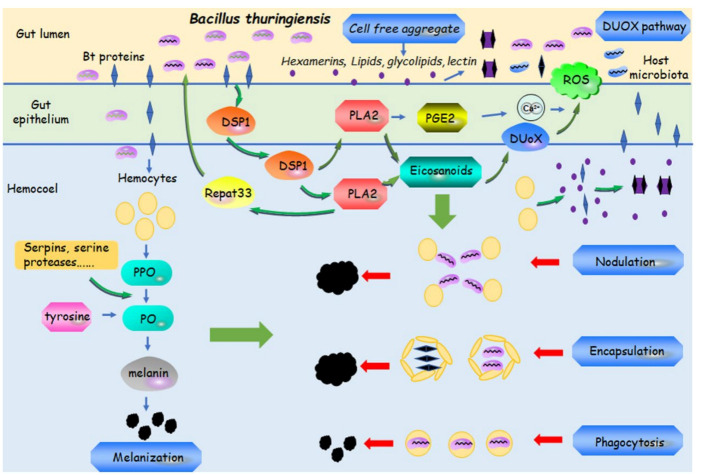
Schematic diagram of the DUOX (dual oxidase) pathway and hemocytes-mediated immune responses to *Bacillus thuringiensis* and/or its toxins. DUOX pathway, phenoloxidase (PO)-dependent melanization, nodulation, encapsulation, phagocytosis and cell free aggregates work in immune responses to *Bt* and/or its toxins. ROS: reactive oxygen species; PPO: prophenoloxidase; DSP1: dorsal switch protein 1; PLA2: phospholipase A2; PGE2: prostaglandin E2.

Melanization is an immune response that is locally triggered in response to systemical microbial invasion following hemocoel or cuticle injury [106]. During melanization, PO is a key enzyme. Active PO is produced from prophenoloxidase (PPO) zymogen by a clip domain serine proteinase (Figure 2) [106]. The PPO-activating proteinase cascade involves the interaction of PRPs, serine protease inhibitors (serpins), serine proteases, PPO-activating proteinase (PAP), and other enzymes (Figure 2) [107,108,109,110]. PO converts tyrosine into the precursors of melanin [111] (Figure 2). Melanin is toxic to parasites, bacteria, fungi, and viruses [106]. It was reported that the expression of *Serpin* and *PAP*s was significantly affected by *Bt* HD73 in *P. xylostella* [112]. Serine proteases and serine protease inhibitors are also highly regulated in *Bt*-susceptible and -resistant *Anticarsia gemmatalis* strains in response to *Bt* [113]. In the midgut of *P. xylostella* larvae, the PPO cascade is induced after infection with *Bt*8010 (the *Bt* strain) [66]. Similar to *Bt* and *Bt* Cry toxins, Cry1Ac induces *Serpin* expression [36], and the upregulated *serpin* gene was involved in the resistance mechanism in *H. armigera* to Cry1Ac [110]. The PPO cascade and highly expressed genes involved in the midgut melanization of *S. exigua* and *S. litura* were also triggered by the Vip3A toxin [89,114]. It was also found that the PPO protein directly binds to Cry1Ah toxin in *O. furnacalis* [115]. As a product of PPO, PO activity could also be induced by *Bt* in *E. kuehniella* and *G. melonella* larvae [27,28,57,116]. PO activity in Cry1Ac-resistant *P. xylostella* was higher than in a Cry1Ac-susceptible strain [117]. Importantly, insect larvae are prone to melanization and develop resistance by increasing melanization in the process of *Bt* infection [26,27,57,118], indicating that melanization may play a key role in the process of resisting infections with *B. thuringiensis* or *Bt* toxins.

### 2.4. Dual Oxidase (DUOX) Pathway against Bt

The DUOX pathway regulates the production of reactive oxygen species (ROS) (Figure 2). ROS are one of the most important molecular effectors in the regulation of insect gut immune responses. Upon infection by a pathogenic bacterium, dorsal switch protein 1 (DSP1) is released to the hemocoel, and then DSP1 activates phospholipase A2 (PLA2) to produce eicosanoids [119,120]. In Lepidopterans, activated PLA2 also increases the level of prostaglandin E2 (PGE2) and subsequently increases Ca^2+^ signal in the gut [119,120]. Meanwhile, eicosanoids activate DUOX [43,121], which promotes the generation of ROS (Figure 2) [119,120]. Infection with *BtK* or *Bt* toxins can induce the upregulation of ROS in the gut lumen [43,122]. ROS are a direct and effective weapon against pathogens [123,124]. In addition, when insects were exposed to *Bt*, a functional binding between the *Bt* toxin and its receptors (cadherin or ABCC) in the midgut epithelium triggered a damage signal. This phenomenon promoted DSP1 release into the hemocoel and then activated PLA2, which upregulated the expression of Repat33 (response to pathogen) inducing the immune response and counteracting *Bt* and *Bt* toxins (Figure 2) [119,120].

### 2.5. Cellular Responses

Cellular responses recruit various hemocytes, leading to nodulation, encapsulation, and phagocytosis (Figure 2) [34]. Nodulation is triggered by infection with small structures or when the initial immune response is insufficient (Figure 2). First, the infection causes the formation of multicellular hemocyte aggregates, and then these aggregates are quickly entrapped. Subsequently, melanization of microorganisms occurs because of the activation of the PO cascade. Finally, these melanized nodules efficiently isolate bacteria from the hemolymph [34,63,125,126]. Encapsulation occurs when the pathogens are relatively larger in size, such as parasites and nematodes. This type of immune response also recruits various hemocytes (Figure 2) [34,125,126]. The invading organisms are killed by reactive cytotoxic products or by asphyxia [127], and melanization involved in the process of encapsulation helps to eliminate the infection from the hemolymph [108,128]. In insects, phagocytosis is performed by a subset of hemocytes in the hemolymph [129] and includes cell recognition, binding, and ingestion of relatively large particles [130] and PPO activation (Figure 2) [131]. Nodulation, encapsulation, and phagocytosis share some common elements, including melanization and ROS, which function in concert to clear pathogens from the hemolymph. *Bt* initiates nodule formation in *S. litura* and *H. zea* larvae, including PO activity and even melanization that are observed after *Bt* infection [116,132]. Doubovskiy et al. [133] and Grizaniva et al. [28] showed that injecting or providing by feeding a spore and crystal mixture of *Bt* with sublethal mortality could increase the phagocytic activity and encapsulation rates in *G. mellonella.* Moreover, live and killed *Bt* bacteria were phagocytosed by macroplasmatocytes and microplasmatocytes [134]. *Sl102* was found to control the nodulation and encapsulation responses. The silencing of *Sl102* by RNAi caused an impairment in the nodulation and encapsulation responses of the hemocytes [16,44,135]. This was associated with a significantly increased susceptibility of the host larvae to the Cry toxin and *Bt* [16,44,135,136].

### 2.6. Cell-Free Aggregates

Another way to diminish the bacterial toxicity in many insects is to produce cell-free aggregates in the lumen and hemolymph (Figure 2). The binding proteins involved in aggregation include hexamerins (a type of inducible immune proteins), lectin, lipids, glycolipids, etc. Hexamerins interact with GalNAc-specific lectin and Cry1Ac to form insoluble aggregates in the hemolymph and midgut lumen of *H. armigera* [137]. In another case, the sequestration of the Cry protein in the insect gut lumen is caused by glycolipids. GalNAc-specific lectins contributed to the interaction between soluble toxin-binding glycoprotein and Cry1Ac, thus forming an insoluble aggregate in Cry1Ac-resistant *H. armigera* [26]. In addition, the aggregation and sequestration of Cry2Ab and Cry1Aa by lipid particles were also found by Ma et al. [138]. These toxin–lipid aggregates are similar to the immune-mediated lipid particle aggregates around lectins [139], which prevent the interaction between specific membrane receptors with *Bt* toxins and cause the inactivation of the Cry protein in the gut lumen. In addition to acting as a PGRP in pathogen recognition, lectin interacts with *Bt* proteins. Some members of the lectin family were upregulated after feeding *Bt* [113,140], thereby diminishing the toxicity in many insects and causing resistance to *Bt* toxin by forming cell aggregates. C-type lectin-20 interacts with alkaline phosphatase 1 (ALP1, a receptor of *Bt* toxins), decreasing the insecticidal activity of Cry toxin in *Aedes aegypti* [141]. Galectins are a family of β-galactoside-binding lectins with similar binding sites as *Bt* toxins, which enable them to bind to *Bt* receptors; this leads to *Caenorhabditis elegans* resistance to Cry5Ba [142]. In *A. aegypti*, galectin-14 can compete with Cry11Aa by binding to the Cry receptor ALP1 [143]. Consistently, galectin-6 was found to interact with ALP1, thus affecting the toxicity of Cry proteins [144].

## 3. Immune Priming

In invertebrates, immune priming is an immune memory-like response. It implies that a previous sublethal exposure to a pathogen confers subsequent immune protection against the same pathogen. Notably, insects could transmit their immune status to the next generation [145,146,147], which may promote the evolution of resistance. As mentioned above, numerous immune genes including PRPs and AMPs are involved in the innate response to *Bt* and are in part responsible for differences between *Bt*-resistant and *Bt*-susceptible strains. Insect resistance to *Bt* formulations or *Bt* crops has been widely reported [17,18,19,20], and the resistance mechanism to *Bt* toxicity is caused by the alteration of host receptors and is associated with increased host immune responses [10,21,22,23,57,148]. Gomez et al. [149] reported that priming with *Bt* resulted in lower mortality in *Tenebrio molitor*. The immune priming of this pest is involved in *Bt*-resistance and can be transmitted to the next generation through a maternal effect [27,150]. Eggert et al. [151] found that the offspring of an immune primed male with increased PGRP expression and PO activity had decreased mortality upon *Bt* bacterial challenge. *G. mellonella* larvae showed an almost 11-fold enhanced resistance to *Bt* compared with a control group after being exposed to *Bt* for over 30 generations [152]. It was reported that amino acids may also help organisms synthesize immune effectors participating in the immune priming response to *Bt* [153]. As a result, immune priming leads to increased resistance to *Bt* [29].

## 4. Inhibition of the Immune Response or of Genes Induced by *Bt* to Improve the Insecticidal Activities of *Bt*

**Figure 3:**
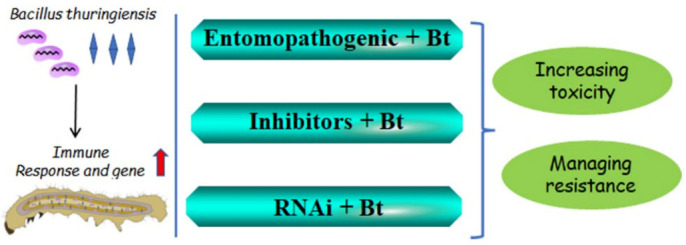
Strategies that inhibit the immune response or genes induced by *Bt* to improve the insecticidal activity of *Bt*. RNAi: RNA interference.

Insect response and resistance to *Bt* or *Bt* toxins indicate that the insect immune system should be a good target for pest control, especially to improve the toxicity to *Bt* or *Bt* toxins and manage insect resistance to *Bt*. Owing to the extensive use of *Bt* crops and *Bt* formulations [1,2,3,4,6], the problem of insect resistance is very serious [17,18,19]. Recently, some new strategies involving *Bt* have been used to improve the insecticidal activity of formulations and prolong the use of *Bt,* avoiding resistance (Figure 3).

### 4.1. Entomopathogenicity Inhibits Insect Immunity to Improve the Toxicity of Bt

Some entomopathogenic inhibitors that suppress the immune system can enhance the insecticidal activities of *Bt* formulations (Figure 3). In the Colorado potato beetle (CPB), co-infection of *C. freundii* and *Bt* can lead to the development of septicemia, and *C. freundii* may affect the humoral and cellular immunity of insects to enhance *Bt* pathogenesis [45]. *Photorhabdus* and *Xenorhabdus* are entomopathogenic bacteria that can synthesize and secrete eicosanoid biosynthesis inhibitors to block the upregulation of ROS in the gut lumen in response to *Bt* infection. In the absence of ROS, the growth of bacteria within the gut lumen increased, leading to enhanced virulence of *Bt* against different target insects, such as *P. xylostella* [43]. Moreover, synergistic toxicity between *Bt* and entomopathogenic fungi was also observed. *Bt* can suppress insect feeding and delay development, thereby increasing the mortality induced by fungus infection [154,155]. The inhibition of cellular immunity in CPB larvae with a sublethal dose of bacteria led to the synergy between the *Bt* sp. *Morrisoni* and the fungus *Metarhizium robertsii* [156]. Consequently, the combination of insect entomopathogenic bacteria or fungi and *Bt* can inhibit insect immunity to improve toxicity.

### 4.2. Inhibitors of Immune Pathways to Improve the Toxicity of Bt

Insect immunity contributes to the defense against *Bt*. The suppression of the insect immune system significantly increased the entomopathogenicity of *Bt* (Figure 3) [157,158,159]. Some commercial PLA2 inhibitors significantly enhance the toxicity of *Bt* by suppressing ROS in the gut lumen [43]. A recombinant immunosuppressive wasp venom protein (rVPr1), derived from the venom gland of *Pimpla hypochondriaca* (an endoparasitic wasp) [160], can suppress the encapsulation response, thus increasing the susceptibility of *Mamestra brassicae* and *Lacanobia oleracea* to *Bt* [161]. Additionally, the inhibition of CaN activity (which may contribute to activate the IMD and Toll pathways to regulate AMP expression) by FK506 or CsA can increase the insecticidal activity of *Bt* toxins in Lepidopteran pests [40,41,162,163,164] and can even be used to manage Cry1Ac resistance in *H. armigera* [42]. Immune inhibitors that improve the insecticidal activity of *Bt* toxins can be used in *Bt* formulations.

### 4.3. RNAi Targeting Immune Genes to Improve the Toxicity of Bt

As aforementioned, *Bt* or *Bt* toxins induce the expression of numerous immune genes in insects. RNAi can be attempted to reduce the expression of these genes and thus theoretically increase the virulence of *Bt*, e.g., by knocking down AMP expression (Figure 3). In *S. exigua* and *G. mellonella*, RNAi-mediated knockdown of gloverin expression enhanced the susceptibility to *Bt* [39,93]. *Sl102* downregulation in *S. littoralis* larvae and feeding bacteria expressing dsRNA enhanced the insecticidal activity of *Bt* kurstaki [44]. RNAi-mediated knockdown of the hemocyte-specific cathepsin L-like cysteine protease gene in *B. mori* can enhance the toxicity of *Bt*-based biopesticides [165]. In addition, a pyramid combining the protection from RNAi and *Bt* toxins is also recommended as a next-generation transgenic to counter insect resistance [166,167]. These immune genes may be candidate targets to enhance the control of pests by *Bt* [38]. RNAi technology can be applied to *Bt* GM crops or used as in formulas with *Bt*.

## 5. Conclusions

*Bt* or *Bt* toxins access insect organism to trigger humoral and cellular immune responses. The humoral response includes the synthesis of AMPs, the activation of the PO system, the generation of ROS, and cellular immune responses (nodulation, encapsulation, and phagocytosis). In addition, cell-free aggregates are widely involved in immune responses to protect insects from *Bt* infection. In addition, insects use immune priming to promote the evolution of resistance to *Bt*. To improve the insecticidal activity of *Bt* formulations and *Bt* crops and also manage insect resistance, it is suggested to explore new entomopathogenicity, immunosuppressant or dsRNA technologies in the future to overcome insect immune protection.

## Data Availability

The data presented in this study are available on request from the corresponding author.
